# Research and Development of a 3D Jet Printer for High-Viscosity Molten Liquids

**DOI:** 10.3390/mi9110554

**Published:** 2018-10-28

**Authors:** Yang Yang, Shoudong Gu, Jianfang Liu, Hongyu Tian, Qingqing Lv

**Affiliations:** School of Mechanical and Aerospace Engineering, Jilin University, No. 5988, Renmin Road, Changchun 130025, China; yangyang17@mails.jlu.edu.cn (Y.Y.); sdgu14@mails.jlu.edu.cn (S.G.); tianhy17@mails.jlu.edu.cn (H.T.); 13861270632@163.com (Q.L.)

**Keywords:** 3D printing, ejection, high-viscosity molten liquid, piezoelectric

## Abstract

Micro-droplet jetting manufacture is a new 3D printing technology developed in recent years. Presently, this new technology mainly aims at ejecting a low-viscosity medium. Therefore, a device for ejecting high-viscosity molten liquid is designed by analyzing the injection principle of high-viscosity molten liquid. Initially, the cooling mechanism is designed to overcome the defect that the piezoelectric stacks cannot operate in high-temperature conditions. Thereafter, the mathematical model of the liquid velocity in the nozzle is derived, and the factors influencing injection are verified by Fluent. Subsequently, a prototype of the jet printer is fabricated, and the needle velocity is tested by the laser micrometer; the relationship between voltage difference and the needle velocity is also obtained. The experimental results matched the theoretical model well, showing that the voltage difference, needle radius, nozzle diameter, and taper angle are closely related to the injection performance of the 3D jet printer. By using a needle with a radius of 0.4 mm, a nozzle with a diameter of 50 μm, a taper angle of 90°, a supply pressure of 0.05 Mpa, and a voltage difference of 98 V, a molten liquid with a viscosity of 8000 cps can be ejected with a minimum average diameter of 275 μm, and the variation of the droplet diameter is within ±3.8%.

## 1. Introduction

3D printing technology is the crystallization of three-dimensional modeling and precision machinery via computer numerical control (CNC) technology; specific materials are stacked layer by layer to create a three-dimensional entity identical to the 3D model [1]. So far, stereo lithography appearance [2,3], selective laser sintering [4,5], fused deposition modeling [6,7], powder material selective bonding technology [8,9], and micro-droplet jetting manufacture [10,11] are relatively developed technologies in the field of 3D printing. Micro-droplet jetting manufacture (MDJM) is a new 3D printing technology developed in recent years. The principle of this technology is based on discrete deposition technology, which sprays liquid through a 3D printing device, controls the trajectory of the droplet ejection via the motion platform, accurately sprays the droplet at a specified position, and gradually accumulates into a three-dimensional model. MDJM has many advantages, such as various types of ejection materials, lower maintenance costs, higher printing precision, and more fine-printed parts, compared with other technologies [12]. This technology is also widely used in biomedical manufacturing, three-dimensional micro-structure manufacturing, the microelectronics industry, micro-spacecraft, and other fields [13].

Scholars have conducted extensive research on the theory and technology of MJDM due to its considerable advantages. Dalian University of Technology built a piezoelectric pulse micropore injection system and sprayed droplets of Sn63Pb37 eutectic alloy [14]. An electric field deflection jetting device was developed by Harbin Institute of Technology and successfully sprayed pure metal tin [15]. South Korea’s Taik-Min Lee et al. designed a piezoelectric molten solder printing system, wherein the diameter and volume of solder balls was 60–150 μm and 0.14–1.8 nL, respectively, and the various tube and rod metal structures were successfully printed [16,17]. However, the current injection apparatus is mainly used for spraying low-viscosity paraffin, alloy materials, and high molecular polymers, and its sprayable viscosity is generally less than 30 cps [18]. Research on the injection of a high-viscosity molten medium in the 3D printing field is limited.

By analyzing the injection principle mechanism of high-viscosity molten liquid, a device for jetting high-viscosity molten liquid, which provides a new method and technology for 3D printing, is designed. The advantages of high precision and fast response of the piezoelectric stacks are applied to jetting high-viscosity molten liquids. Initially, the cooling mechanism is designed to overcome the defect that the piezoelectric stacks cannot operate in high-temperature conditions. Thereafter, the mathematical model of the liquid velocity in the nozzle is derived, and the influencing factors on injection are verified by Fluent. Subsequently, the prototype of the jet printer is fabricated, the displacement of the needle is tested by a laser micrometer, and the influence of voltage on the needle velocity is obtained. Finally, the experimental results corroborate that the voltage difference, needle radius, nozzle diameter, and taper angle are closely related to the injection performance of the jet printer.

## 2. Principle of the 3D Jet Printer

### 2.1. Structure of the Device

**Figure 1:**
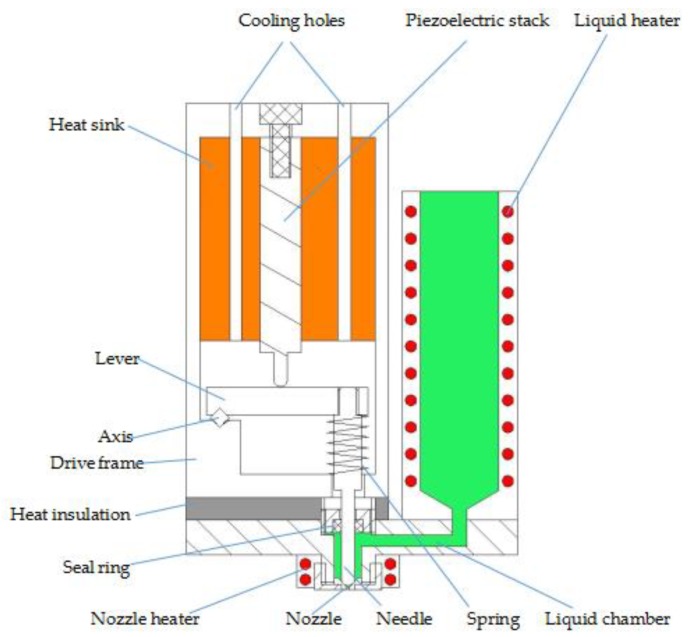
Structure of the jet printer.

Figure 1 shows the structure of the jet printer, which comprises the piezoelectric stack, drive frame, lever, heat insulation, heat sink, heater, needle, and nozzle. The piezoelectric stack is sintered from many ceramic sheets. It is the power source of the device for its high-frequency vibration after receiving the square wave signal. The jet printer mainly uses a lever to amplify the displacement of the piezoelectric stack, and the needle moves together with the lever. The liquid chamber is heated by the heater to melt viscous liquid, and the heat will be transferred to the piezoelectric stack, which also generates heat when working. This phenomenon will cause damage to the piezoelectric stack if the surface temperature is too high. Therefore, insulation is used to protect the piezoelectric actuator from the heat induced by the heater. Moreover, the heat sink and cooling holes are used to dissipate the heat generated by the piezoelectric stack.

**Figure 2:**
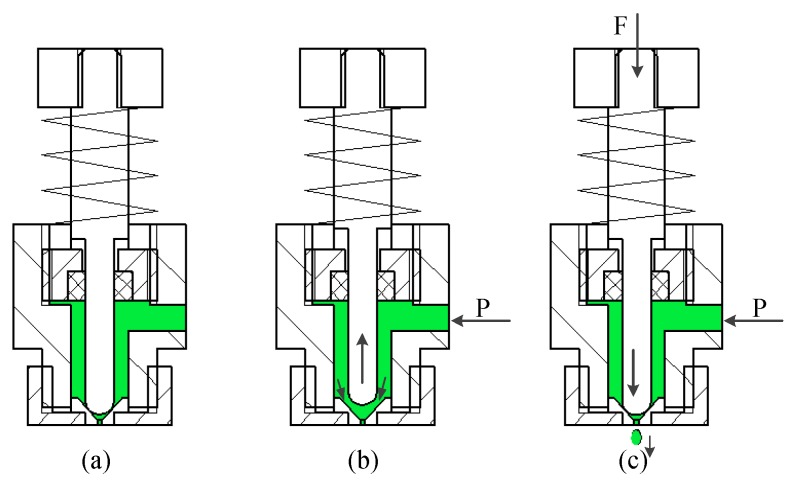
Jetting principle. (**a**) Normal state; (**b**) needle upward movement; (**c**) needle downward movement.

Figure 2 shows that the jetting principle of the jet printer can be divided into the following three steps:

1. In the normal state, the piezoelectric stack is powered on, and the needle and the nozzle are in a close-fit state with the role of sealing, which can effectively prevent the fluid in the chamber from flowing out through the nozzle orifice (Figure 2a).

2. The needle separates from the nozzle under the restoring force of the spring when the piezoelectric stack is powered off. The fluid in the chamber is transported to the gap between the needle and nozzle under pressure (Figure 2b).

3. The needle moves down while an electric signal is applied to the piezoelectric stack, and instantaneous high pressure can be formed between the needle and nozzle. The high pressure generated will force the fluid at the nozzle orifice to overcome the fluid force, thereby realizing the injection (Figure 2c).

### 2.2. Thermodynamic Analysis

Piezoelectric stacks can convert electrical energy into mechanical energy. However, a large amount of heat is generated when operating at high frequencies because of the internal friction during operation. The amount of heat generated can be expressed as [19]:

(1) $$\;P=\;\frac{\pi }{4}\tan\delta \cdot f\cdot C\cdot U_{\mathrm{PP}}{}^{2}\;$$

where *P* is the heat value generated, tanδ is the dielectric loss coefficient, *f* is the driving frequency, *C* is the equivalent capacitance of the piezoelectric stack, and $U_{\mathrm{PP}}$ is the peak-to-peak value of applied voltage.

To ensure the stable working state of the piezoelectric stack, the long-term working temperature should be less than 50% of the Curie temperature [20] (i.e., the temperature of the piezoelectric stack should be less than 175 °C when working). During operation, both the heater and heat generated by the piezoelectric stack itself can increase the temperature of the piezoelectric stack. Therefore, the stack must be properly cooled to prevent it from being damaged by overheating.

Thermodynamic simulation is then carried out, and a proper structure is designed to reduce the heat of the piezoelectric stack when operating at high frequency. The model of the jet printer is imported into Ansys Workbench (V14.5) (Ansys, Inc., Canonsburg, PA, USA), its main parts are composed of 45-section structural steel, the nozzle and liquid heater temperature are set to 180 °C and 120 °C separately, the ambient temperature is set to 25 °C, the convection coefficient is set to 5 W/(m^2^·k), and the piezoelectric stack heating coefficient is set to 2.04 × 10^6^ W/m^3^.

**Figure 3:**
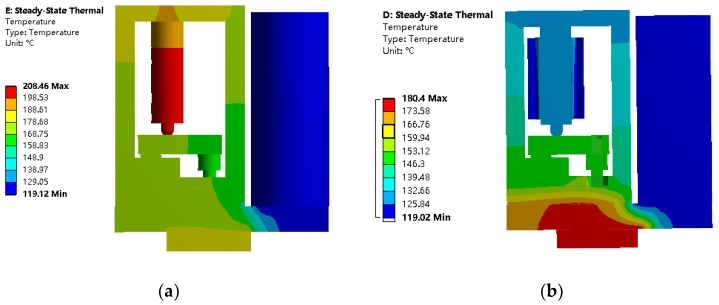
Thermodynamic simulation cloud. (**a**) Operating without cooling mechanism; (**b**) operating with cooling mechanism.

The thermodynamic simulation of the jet printer without the cooling mechanism is shown in Figure 3a. The temperature distribution of the piezoelectric stack is between 188 °C and 208 °C. When working in this condition, the piezoelectric stack will incur damage. As shown in Figure 3b, a layer of PEEK insulation is added between the drive frame and liquid chamber to reduce the heat transferred by the heater. A layer of heat sink is placed around the piezoelectric stack, and air flows through its interior. The temperature distribution of the piezoelectric stack is between 125 °C and 132 °C, and the temperature of the piezoelectric stack is reduced to a suitable value.

The thermodynamic analysis of the jet printer reveals that the cooling mechanism can be designed to effectively reduce the temperature during the operation of the piezoelectric stack. Thus, even the limit condition can be satisfied.

### 2.3. Theoretical Analysis of Jetting

Many scholars have analyzed the principle of the droplet ejection. The inertial force obtained by the droplet must overcome the viscous drag and surface tension. The fluid velocity must exceed a critical value for the adhesive solution to achieve injection. A large fluid velocity allows the droplet to gain a large kinetic energy, making it easy for the fluid to overcome its own viscous force and spray out, which results in large fluid viscosity.

**Figure 4:**
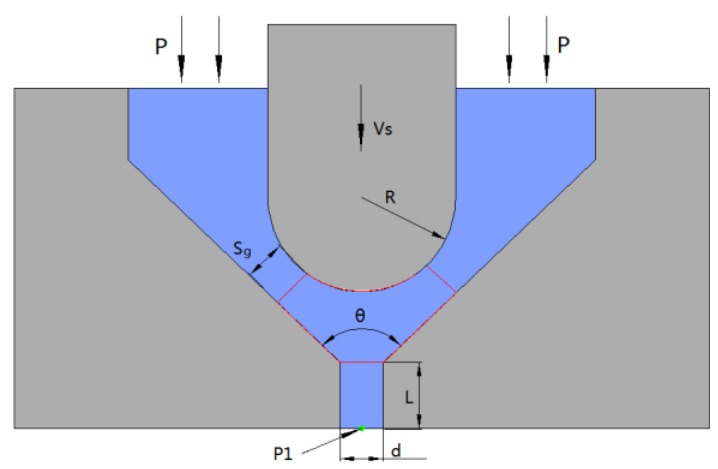
Geometric model of the needle and nozzle.

Owing to the high viscosity of the fluid, all the fluid flow is assumed to be in the laminar regime. Figure 4 shows a geometric model of the needle and nozzle. The model is used to analyze the state of liquid movement during the collision of the needle with the nozzle. During the impact of the needle against the nozzle, a portion of the molten liquid between the needle and the nozzle will be ejected from the nozzle orifice, and the remaining liquid will flow back to the liquid storage chamber. The law of weight conservation shows the following:

(2) $$\;Q=\;Q_{g}+Q_{h}\;$$

where *Q* is the reduced flow between the needle and the nozzle when the needle moves downward, $Q_{g}$ is the flow rate of the returning liquid, and $Q_{h}$ is the flow rate in the nozzle orifice.

When the needle moves downward at velocity *v_s_*, the reduced flow between the needle and the nozzle is

(3) $$\;Q=\;\pi v_{s}R^{2}\cos^{2}\frac{\theta }{2}\;$$

where *R* is the needle radius and θ is the nozzle taper angle.

When the needle nearly hits the nozzle, the gap between the needle and the nozzle is infinitely close to 0, ${}_{S_{g\to 0}}^{Q_{g}}=0$; therefore,

(4) $$\;Q=Q_{h}=\frac{\pi d^{2}}{4}V\;$$

where $S_{g}$ is the gap between the needle and the nozzle, *d* is the diameter of the nozzle orifice, and *V* is the velocity of the droplet in the nozzle orifice.

Therefore, when the needle hits the nozzle, the velocity of the droplet in the nozzle orifice can be determined by Equations (3) and (4).

(5) $$\;V=\frac{4V_{s}R^{2}\cos^{2}\frac{\theta }{2}}{d^{2}}\;\;$$

Equation (5) shows that the ejection velocity of the droplet is influenced by the needle velocity, needle radius, nozzle diameter, and taper angle.

### 2.4. Analysis of Jetting

According to the theoretical analysis of jetting, the velocity of the liquid in the nozzle orifice is mainly related to the impact velocity of the needle, the structural parameters of the needle, and the nozzle. Fluent (V6.3) (Ansys, Inc., Canonsburg, PA, USA) is used to simulate and analyze the influence of the aforementioned parameters on the flow rate in the nozzle orifice.

The geometric model shown in Figure 4 is introduced to Fluent for simulation analysis. Polyurethane molten liquid with a temperature of 120 °C is used as the simulation object. The density is set to 1300 kg/m^3^, the viscosity is set to 8000 cps, the surface tension coefficient is set to 20 mN/m, and the yield stress is set to 150 Pa in the simulation software. Point P1 is set at the outlet of the nozzle orifice to monitor the velocity of the flow. The inlet and outlet pressures are set to 2 × 10^5^ Pa and 1.01 × 10^5^ Pa, respectively. The needle radius *R* and velocity *V_s_* are set to 0.75 mm and 1.5 m/s, the nozzle diameter *d* and taper angle θ are set to 100 μm and 90°, the length of the nozzle tip *L* is set to 0.3 mm as boundary conditions.

**Figure 5:**
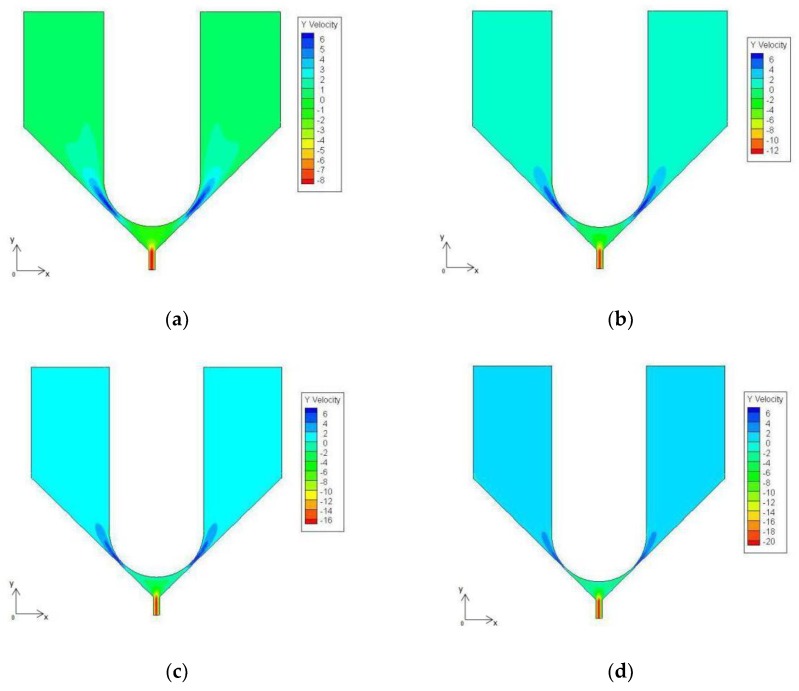
Velocity cloud at different times. (**a**) t1 = 2 × 10^−5^ s; (**b**) t2 = 4 × 10^−^^5^ s; (**c**) t3 = 6 × 10^−^^5^ s; (**d**) t4 = 8 × 10^−^^5^ s.

According to the simulation, as the needle impacts, the velocity of the molten liquid at point P1 at different times is shown in Figure 5. The figure shows that, during the downward impacting of the needle, the liquid in the gap between the nozzle and the needle flows upward, the liquid in the nozzle orifice flow downwards, and the flow velocity at point P1 gradually becomes large.

Thereafter, the flow rate at point P1 will be simulated at the instant of the needle striking the nozzle (at t4) to analyze the influence of these parameters on the injection.

#### 2.4.1. Simulation of the Needle Velocity

**Figure 6:**
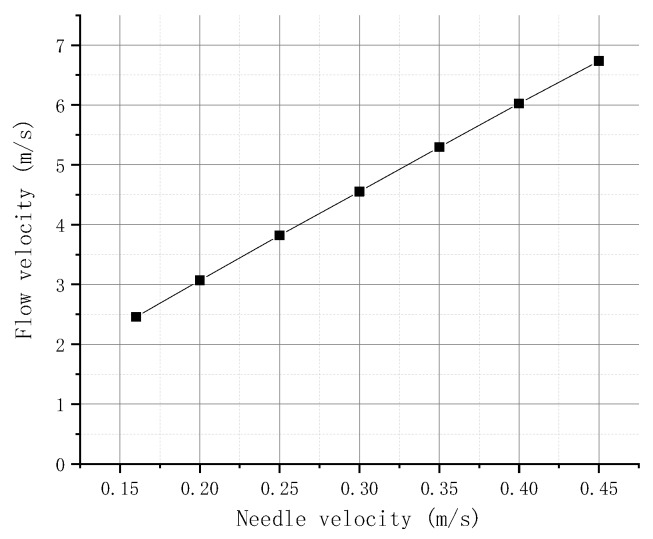
Flow velocity in different needle velocities.

The velocity of the needle is set to 0.16, 0.2, 0.25, 0.3, 0.35, 0.4, and 0.45 m/s. Figure 6 exhibits the relationship between the flow velocity of the molten liquid at point P1 and the needle velocity obtained by simulation. The figure shows that, when the needle hits the nozzle, the flow velocity at the P1 point gradually increases with the needle velocity and is basically linear. This finding can be attributed to the increase in needle velocity, which increases the reduced flow rate between the needle and the nozzle in unit time, resulting in large flow velocity in the nozzle.

#### 2.4.2. Simulation of the Needle Radius

**Figure 7:**
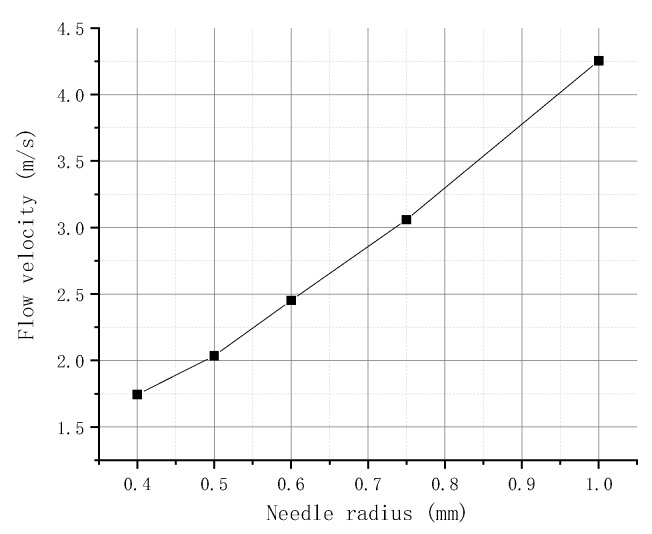
Flow velocity in different needle radii.

The radius of the needle is set to 0.4, 0.5, 0.6, 0.75, and 1 mm. Figure 7 depicts the relationship between the flow velocity of the molten liquid at point P1 and the needle radius obtained by simulation. The figure shows that, when the needle hits the nozzle, the velocity of the flow at the P1 point gradually increases with the needle radius. This finding is due to the large volume of liquid wrapped between the needle and the nozzle when the needle radius is increased. The pressure generated between the needle and the nozzle becomes large when the needle hits the nozzle. More liquid also flows out in unit time, and the flow velocity in the nozzle becomes large.

#### 2.4.3. Simulation of the Nozzle Diameter

**Figure 8:**
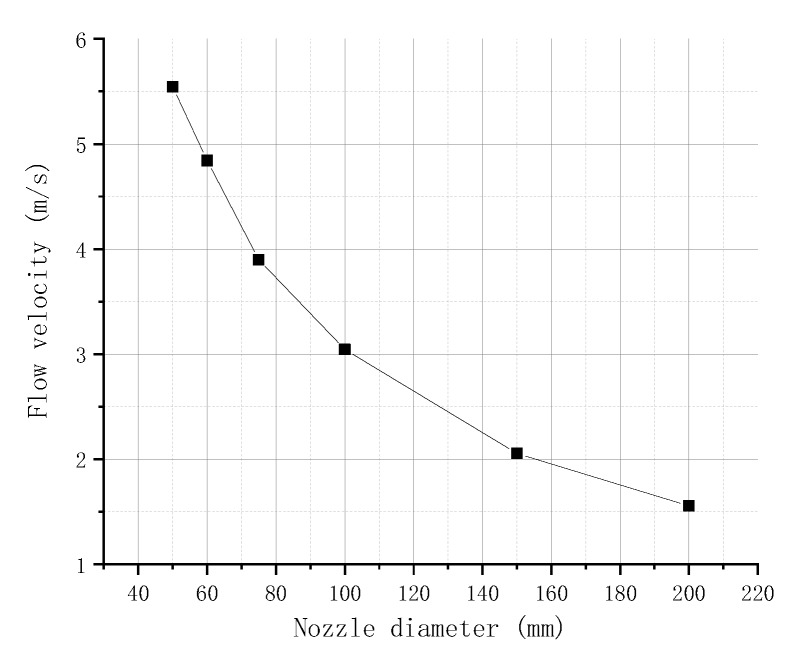
Flow velocity in different nozzle diameters.

The diameter of the nozzle is set to 50, 60, 75, 100, 150, and 200 μm, and the relationship between the flow velocity of the molten liquid at point P1 and the nozzle diameter obtained by simulation is shown in Figure 8. The figure shows that, when the needle hits the nozzle, the flow velocity at the P1 point gradually decreases with the increase in the nozzle diameter. This finding can be attributed to the increase in the nozzle diameter, which decreases the resistance of the molten liquid, resulting in small pressure generated by the needle impact; accordingly, the velocity of the flow in the nozzle is reduced.

#### 2.4.4. Simulation of the Nozzle Taper Angle

**Figure 9:**
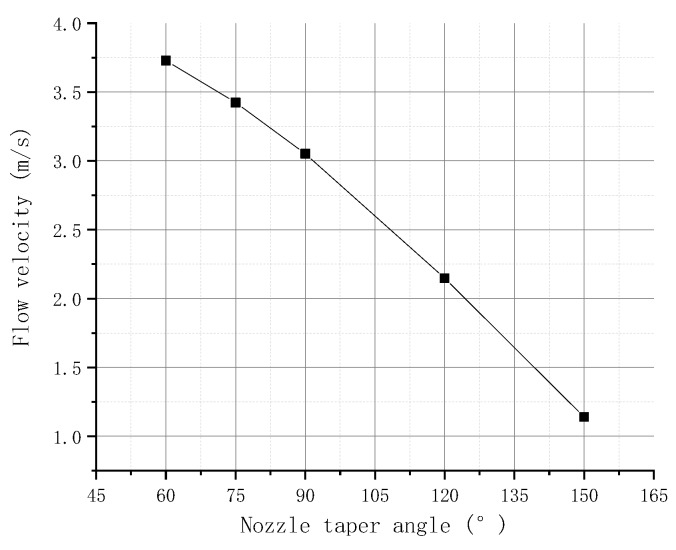
Flow velocity in different nozzle taper angles.

The taper angle of the nozzle is set to 60°, 75°, 90°, 120°, and 150°. Figure 9 shows the relationship between the flow velocity of the molten liquid at point P1 and the nozzle taper angle obtained by simulation. The figure shows that, when the needle hits the nozzle, the velocity of the flow at the P1 point gradually decreases with the increase in the nozzle taper angle. This finding is due to the small pressure generated by the impact of the needle when the taper angle of the nozzle is large. Moreover, the molten liquid flowing out from the nozzle orifice and the gap is reduced in unit time, and the velocity of the flow in the nozzle is reduced accordingly.

Fluent is used to demonstrate the effect of the needle velocity and the radius, and the nozzle on the injection of the molten liquid in jetting. The injection velocity of the molten droplet is positively correlated with the velocity and the radius of the needle and negatively correlated with the diameter and taper angle of the nozzle.

## 3. Experiment and Discussion

### 3.1. Experiment on the Needle Velocity

**Figure 10:**
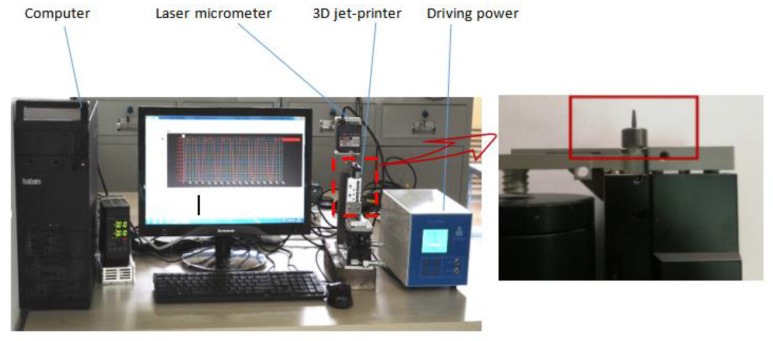
Test bench for needle movement.

As indicated above, the needle velocity has an important influence on the injection of molten liquid. However, the velocity of the needle in the jet printer is controlled by the driving power of the piezoelectric stack. A test bench for needle movement is built to obtain the relationship between the needle velocity and the voltage difference generated by the driving power. Figure 10 exhibits that the test bench is composed of a driving power, a 3D jet printer, a laser micrometer (Keyence, LK-H020, Keyence Corporation, Osaka, Japan), and a computer. The piezoelectric stack vibrates under the driving power and drives the needle to move through the lever amplification system.

**Figure 11:**
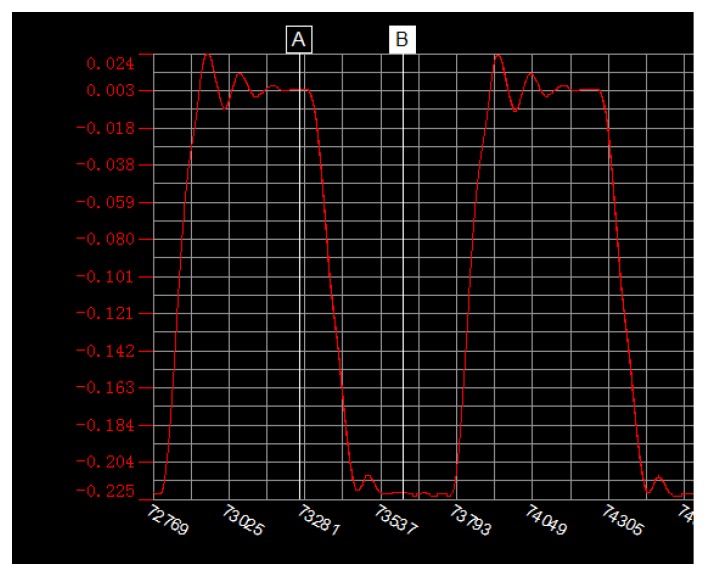
Motion characteristic curve of the needle.

Figure 11 shows the motion characteristic curve of the needle when the voltage difference is 100 V and the high and low voltage times are both 1.5 ms. The ordinate represents the relative displacement of the needle and the abscissa represents the points collected by the laser micrometer (which can be converted to time). The rising edge represents the upward movement of the needle, and its movement time can be calculated by the chart. Cursor A is at a high level and cursor B is at a low level. The difference between the ordinates of the cursor A and B is the absolute displacement of the needle. The needle velocity can be obtained via absolute displacement divided by the time of the rising edge.

**Figure 12:**
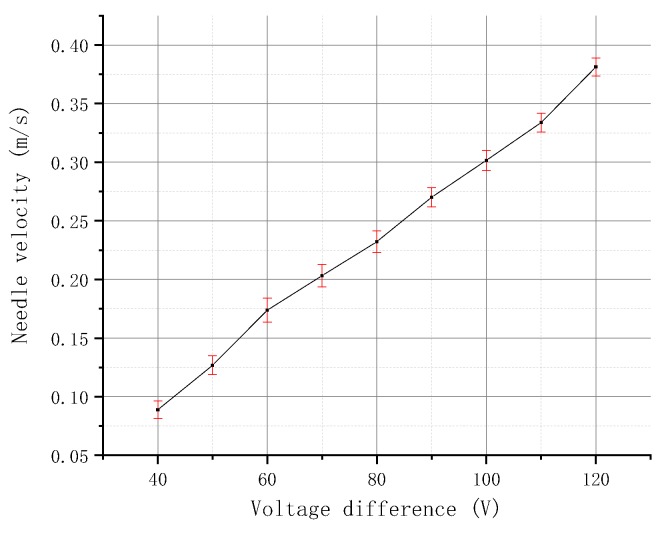
Relationship between needle velocity and voltage differences.

Then, the experiment of the needle velocity will be carried out in different voltage differences. Figure 12 depicts that the velocity curve of the needle at different voltage difference is obtained after several measurements. The figure also shows that, as the voltage difference increases, the needle velocity increases accordingly.

Given that the needle velocity is positively correlated with the voltage difference, the following experiments use the voltage difference instead of the needle velocity as a variable and always set the high and low voltage time to 1.5 ms.

### 3.2. Experiment on Influencing Factors of Injection

Through the previous theoretical analysis and the testing of the needle velocity, spraying molten droplets of high viscosity is possible by setting different needle radii, nozzle diameters, and taper angles and by adjusting the driving voltage. Therefore, the experiments will be separately performed for each parameter, and its effect on spraying high-viscosity molten liquid will be verified.

**Figure 13:**
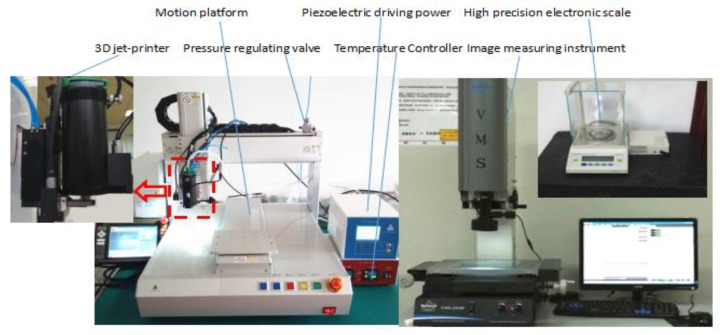
Jet printing test bench.

The medium used in this test is polyurethane (Loctite, Düsseldorf, Germany, model: 3542), which is solid at normal temperature and has a viscosity of 8000 cps after heating to 100 °C. Figure 13 shows that the experimental system includes a 3D jet printer, piezoelectric driving power, motion platform, high-precision electronic scale (Sartorius, BT125D, Goettingen, Germany, range: 120 g, resolution: 0.01 mg), image measuring instrument (Wanhao, VMS-1510F, Odessa, FL, USA, precision: 2 μm), and a pressure supply system.

The motion platform drives the 3D jet printer according to the set program. When the specified position is reached, the motion platform sends a signal to the piezoelectric driving power. Thereafter, the driving power sends a pulse signal and drives the piezoelectric stack, and the droplets are ejected from the printer onto the substrate.

In the following experiments, the liquid and nozzle heater are set to 100 °C and 120 °C, respectively, and the air pressure supply is set to 0.2 MPa in the whole process. The weight of the droplets in this paper is based on the injection of 1000 droplets, the total weight is measured by a precision electronic scale, and the average weight of a single point is obtained by calculation. The diameter of a droplet that is cooled and solidified on the substrate is measured by an image measuring instrument.

#### 3.2.1. Experiment on the Voltage Difference

**Figure 14:**
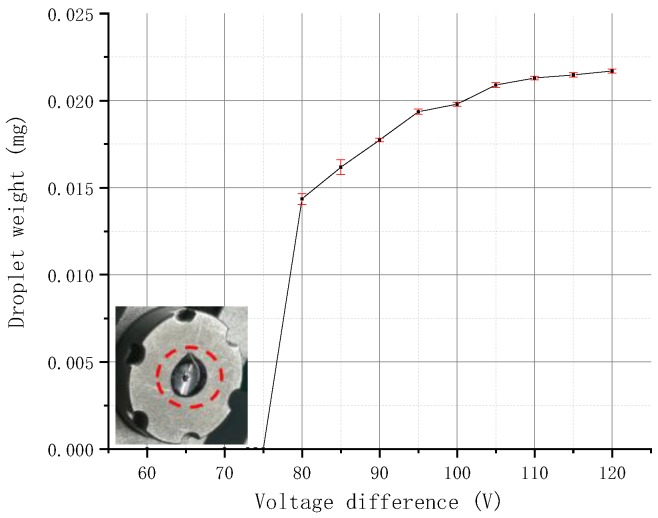
Relationship between voltage difference and droplet weight.

The needle velocity can be controlled by adjusting the different voltage parameters. The selected nozzle and needle have a diameter of 100 μm, a taper angle of 90°, and a radius of 0.75 mm, respectively. Different voltage differences are set for jetting experiments, and the droplet weight is measured and recorded. The relationship between the droplet weight and the voltage difference is plotted (Figure 14).

Figure 14 shows that, when 75 V < voltage difference < 120 V, the weight of the molten droplet is positively correlated with the voltage difference. When the voltage difference is large, the moving velocity of the needle and the inertia force obtained by the droplet will also be large, and the weight of the ejected droplets will increase accordingly. When the voltage difference <75 V, the molten droplets cannot be detached from the nozzle to achieve jetting but are deposited at the nozzle orifice. The ejection cannot be formed because the inertial force of the droplet cannot overcome its own viscous resistance when the voltage difference is reduced to a certain value.

#### 3.2.2. Experiment on the Needle Radius

The nozzle with a diameter of 100 μm and a taper angle of 90° is selected, and the voltage difference is set to 100 V. The needles with radii of 0.4, 0.5, 0.75, and 1 mm are selected for the jetting experiment. The weight of the sprayed droplets is then measured.

**Figure 15:**
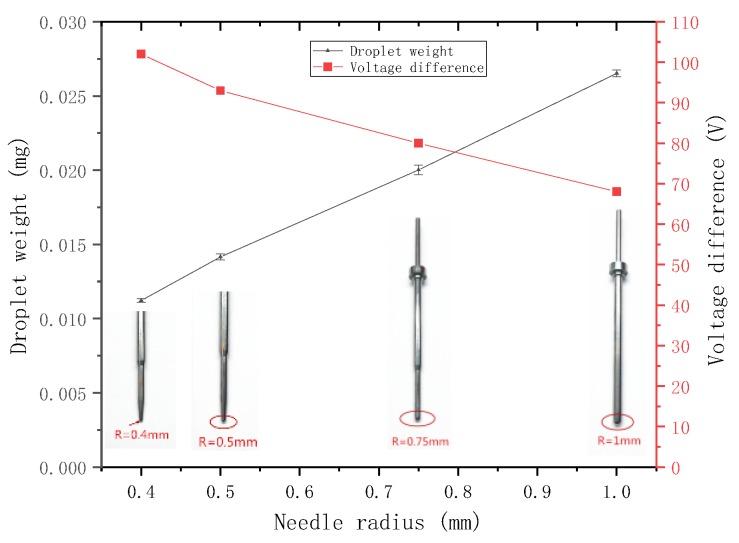
Relationship between the droplet weight, the minimum voltage difference, and the needle radius.

Then, the other parameters are kept unchanged for another experiment, the minimum voltage difference that can form a stable ejection under each needle is adjusted and recorded. The relationship between the droplet weight, the minimum voltage difference that can form a stable ejection, and the needle radius, is plotted (Figure 15).

The figure shows that, when the other parameters are the same, a large needle radius results in sprayed droplets with large weight and requires a small voltage difference to form a stable ejection, that is, the ability to jet is strong. The experimental conclusions are consistent with those obtained from the previous analysis.

#### 3.2.3. Experiment on the Nozzle Diameter

The needle with the radius of 0.75 mm is selected, and the voltage difference is set to 100 V. The nozzles with the diameter of 50, 75, 100, and 150 μm and taper angle of 90° are selected for jetting experiment. The weight of the sprayed droplets is then measured.

**Figure 16:**
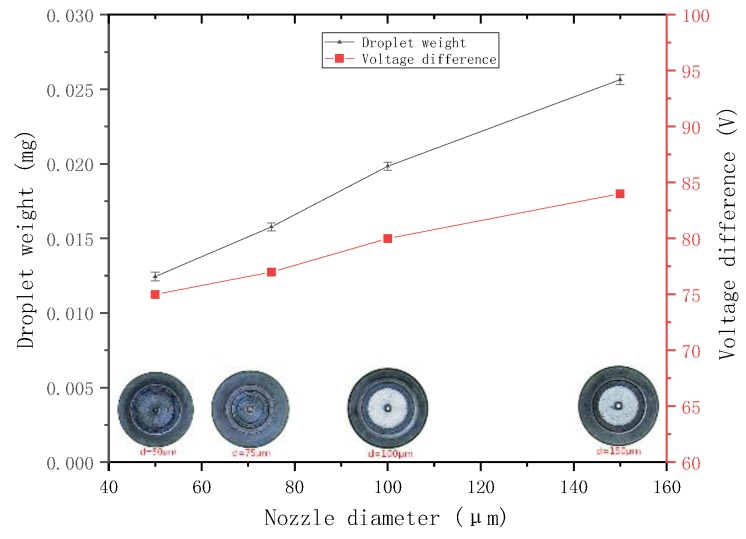
Relationship between the droplet weight, the minimum voltage difference, and the nozzle diameter.

Then, the other parameters are kept unchanged for another experiment, the minimum voltage difference, which can form a stable ejection under each nozzle, is adjusted and recorded. The relationship between the droplet weight, the minimum voltage difference that can form a stable ejection, and the nozzle diameter, is plotted (Figure 16).

The figure shows that, when the other parameters are the same, a large nozzle diameter results in sprayed droplets with large weight and requires a large voltage difference to form a stable ejection, that is, the ability to jet is weak. The experimental conclusions are consistent with those obtained from the previous analysis.

#### 3.2.4. Experiment on the Nozzle Taper Angle

The needle with the radius of 0.75 mm is selected, and the voltage difference is set to 100 V. The nozzles with taper angles of 60°, 90°, and 120° with a diameter of 75 μm are set for jetting experiment. The weight of the sprayed droplets is measured and recorded.

**Figure 17:**
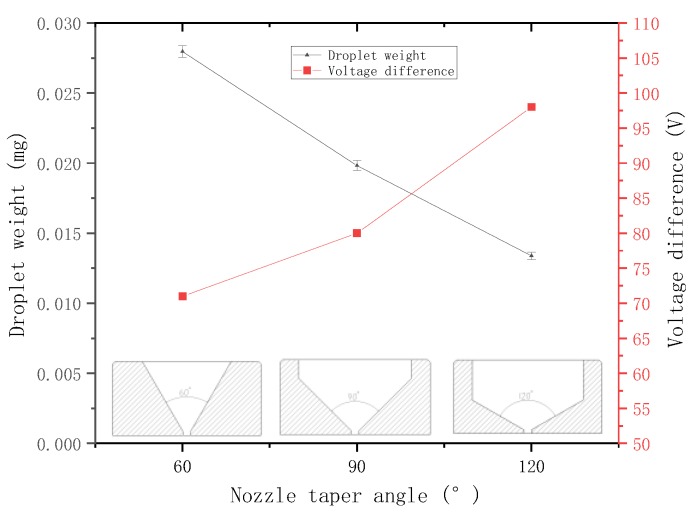
Relationship between the droplet weight, minimum voltage difference, and nozzle taper angle.

Then, the other parameters are kept unchanged for another experiment, the minimum voltage difference that can form a stable ejection under each nozzle is adjusted and recorded. The relationship between the droplet weight, the minimum voltage difference that can form a stable ejection, and the nozzle taper angle is plotted (Figure 17).

The figure shows that, when the other parameters are the same, a large nozzle taper angle results in sprayed droplets with small weight and requires a large voltage difference to form a stable ejection, that is, the ability to jet is weak. The experimental conclusions are consistent with those obtained from the previous analysis.

### 3.3. Minimum Droplet and Consistency Analysis

The minimum molten droplet determine the injection resolution of the jet printer. According to the previous analysis and experiment, the dimension of the droplet is affected by the voltage difference, needle radius, nozzle diameter, and taper angle. These factors will also affect the injection capability of the jet printer. Table 1 shows the specific relationship (“↑” represents positive correlation, and “↓” represents negative correlation).

The minimum droplet can only be obtained by selecting the optimum configuration and adjusting the appropriate parameters. In the next experiment, 10 droplets will be ejected under each set of configuration and parameters in Table 2, every droplet will be measured and the droplet diameter ultimately obtained is the average value via calculation.

Table 2 shows that the needle with a radius of 0.4 mm, the nozzle with a taper angle of 90° and a diameter of 50 μm, a minimum voltage difference of 98 V, and a supply pressure of 0.05 MPa can obtain a minimum molten droplet, and the minimum average diameter is 275 μm.

**Figure 18:**
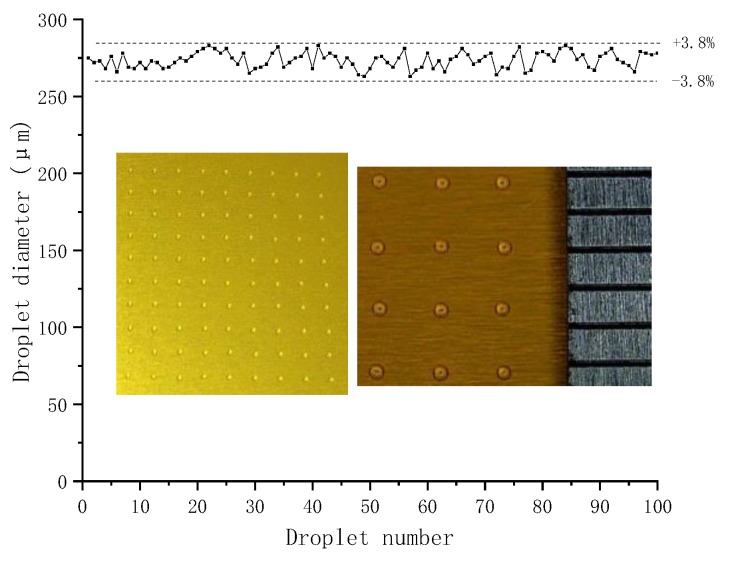
Droplet diameter distribution.

Then, the consistency of the droplets will be analyzed. 100 droplets on the substrate were achieved with the configuration that are able to get the minimum droplet. Figure 18 shows a 10 × 10 droplet array with an average diameter of 275 μm, and the variation of the droplet diameter was within ±3.8%.

**Figure 19:**
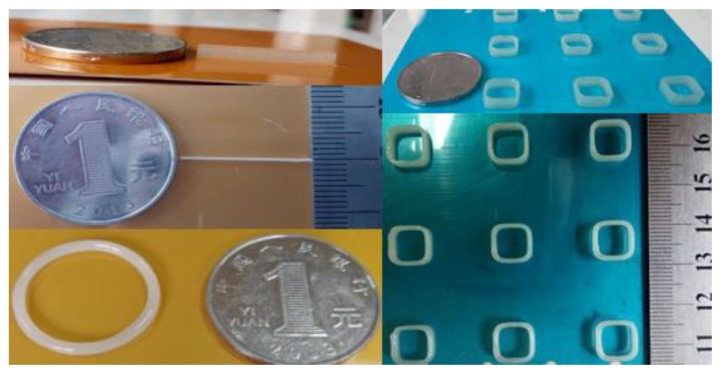
Printed simple model.

The simple patterns shown in Figure 19 are all printed on the basis of the minimum droplets by the 3D jet printer herein.

## 4. Conclusions

A piezoelectric 3D jet printer for high-viscosity molten liquid was newly devised in this study. Through theoretical analysis, simulation analysis, and experimental research, the influence of the needle velocity, needle radius, nozzle diameter, and taper angle on the jetting performance of high-viscosity molten liquid was verified. This study found the following:

1. The defect that the piezoelectric stacks cannot operate in high-temperature conditions can be solved by the cooling mechanism designed in this paper.
2. The experiments verified that the velocity of the needle is positively correlated with the voltage difference of the piezoelectric stacks.
3. Through simulation analysis and experimental research, the ejection capacity of the jet printer is positively correlated with the velocity and the radius of the needle and negatively correlated with the diameter and taper angle of the nozzle.
4. Through experimental comparison, by using a needle with a radius of 0.4 mm, a nozzle with a diameter of 50 μm, a taper angle of 90°, a supply pressure of 0.05 Mpa, and a voltage difference of 98 V, a molten liquid with a viscosity of 8000 cps can be sprayed with the minimum average droplet diameter of 275 μm, and the variation of the droplet diameter was within ±3.8%.

In this study, the experimental medium used is a type of polyurethane. For the next step, the focus should be on the effect of other high-viscosity molten liquids that have not been used for jetting in the 3D printing field before.

## Figures and Tables

**Table 1:** Relationship between influencing factors, droplet weight, and injection capacity.

Influencing Factor	Droplet Weight	Injection Capacity
voltage difference	↑	↑
Needle radius	↑	↑
Nozzle diameter	↑	↓
Nozzle taper angle	↓	↓

**Table 2:** Droplet diameter in different configurations and parameters.

Serial Number	Needle Radius (mm)	Taper Angle (°)	Nozzle Diameter (μm)	Minimum Voltage Difference (V)	Supply Pressure (MPa)	Droplet Diameter (μm)
1	0.4	60	50	90	0.05	295
2	0.4	90	50	98	0.05	275
3	0.4	120	50	120	0.05	-
4	0.5	120	50	112	0.05	305
